# Three-dimensional transvaginal ultrasonography in the evaluation of diminished ovarian reserve and premature ovarian failure

**DOI:** 10.12669/pjms.39.3.7372

**Published:** 2023

**Authors:** Qian Chen, Lu Sun, Jian Huang, Fan Huang, Shuang Guo, Yiqing Chai

**Affiliations:** 1 Qian Chen Department of Ultrasound, Tianjin Central Hospital of Gynecology Obstetric, Tianjin 300100, P.R. China; 2 Lu Sun Department of Gynecology, Tianjin Central Hospital of Gynecology Obstetric, Tianjin 300100, P.R. China; 3 Jian Huang Department of Ultrasound, Tianjin Central Hospital of Gynecology Obstetric, Tianjin 300100, P.R. China; 4 Fan Huang Department of Ultrasound, Tianjin Central Hospital of Gynecology Obstetric, Tianjin 300100, P.R. China; 5 Shuang Guo Department of Ultrasound, Tianjin Central Hospital of Gynecology Obstetric, Tianjin 300100, P.R. China; 6 Yiqing Chai Department of Ultrasound, Tianjin Central Hospital of Gynecology Obstetric, Tianjin 300100, P.R. China

**Keywords:** Three-dimensional transvaginal ultrasonography, Diminished ovarian reserve, Premature ovarian failure

## Abstract

**Objective:**

To explore the applicability of three-dimensional transvaginal ultrasonography (3D-TVS) in the evaluation of diminished ovarian reserve (DOR) and premature ovarian failure (POF).

**Methods:**

One hundred and twenty female patients, who received 3D-TVS in our hospital from January 2020 to March 2022, were included in the study. Based on sex hormone examination, 25 cases were DOR (DOR-group), 32 cases were POF (POF-group) and 63 cases had normal ovarian function (Normal-group). The 3D-TVS quantitative examination results of the three groups of patients were analyzed and compared.

**Results:**

There was no significant difference between the DOR-group and POF-group regarding antral follicles count (AFC), ovarian volume (OV), vascularization index (VI), vascularization flow index (VFI) and flow index (FI) of left and right ovaries (p>0.05). Compared with the Normal-group, the 3D-TVS examination indexes of the DOR-group and POF-group were significantly lower, and the 3D-TVS examination results of the POF-group were significantly lower than those of the DOR-group (p<0.05). Using sex hormone examination as the gold standard, the diagnostic specificity of 3D-TVS for DOR was 80%, and the sensitivity and accuracy were 90% and 88% respectively; The diagnostic specificity of POF was 87.5%, the sensitivity and accuracy were 95.8% and 93.8% respectively.

**Conclusion:**

3D-TVS can provide scientific guidance for the clinical diagnosis and evaluation of DOR and POF.

## INTRODUCTION

Diminished ovarian reserve (DOR) refers to the decrease in number of follicles contained in the ovaries of mature women with age.^1^ Aging also results in a gradual decline in the development and production capacity of fertilizable oocytes.^1^,^2^ If DOR is not managed in time, the condition will continue to develop and deteriorate, and eventually develop into premature ovarian failure (POF), which refers to amenorrhea in women before the age of 40 due to ovarian failure.^3^ Early detection of DOR and strengthening of treatment are the keys to POF prevention.^4^,^5^ At this stage, endocrine hormone detection and ultrasonographic imaging are often used for DOR detection in clinic. Ultrasonographic examination is widely recognized and promoted due to its noninvasive, simple operation and dynamic imaging advantages.^6^,^7^

Specifically, transvaginal three-dimensional ultrasound can obtain more reliable information about sinus follicular number and ovarian volume, and can clearly display the small structure of the ovary, which is conducive to further understanding its blood perfusion.^8^ Some studies have pointed out that 3D-TVS can effectively detect the blood flow of ovarian stroma through three-dimensional reconstruction technology, and can provide a more scientific and reliable reference basis for clinical analysis of ovarian blood supply.^9^,^10^ This study analyzed and compared the 3D-TVS results of DOR, normal ovarian function and POF, and analyzed the applicability of this technology in evaluating DOR and POF.

## METHODS

One hundred and twenty female patients with suspected decreased ovarian reserve function who received 3D-TVS in our hospital from January 2020 to March 2022, were selected for the study. Based on sex hormone examination, 25 cases were deemed DOR, and set as the DOR-group, 32 cases were deemed POF and set as the POF-group and 63 cases were deemed normal ovarian function and set as the Normal-group.

### Inclusion criteria:

- Patients must have had previous blood samples collected during the early follicular stage (no time limit for amenorrhea).
- Follicular estrogen (FSH) level must exceed 40 IU/L at least twice (the interval between two examinations was more than a month), and estradiol (E2) level was less than 73.2pmol/L, which was included in POF-group (32 cases).
- FSH examination results were in the range of 10-40IU/L, FSH / luteinizing hormone (LH) was more than 3.6IU/L, and E2 was less than 43.9pmol/l, which were included in DOR-group (25 cases),
- FSH test results less than 10 IU/L were included in the Normal-group (63 cases).

### Exclusion criteria:

- Received hormone therapy in recent three months.
- Received pelvic surgery.
- Endocrine diseases.
- Ovarian cyst, polycystic ovary syndrome or other ovarian and uterine organic diseases.
- Follicle diameter exceeds 10mm.
- The display of one or both ovaries is vague, and the volume of collected ovaries is not ideal, so software analysis cannot be carried out.

### Ethical approval:

This study was approved by the ethics committee of our hospital (Ref.: TF22122; Date: 2022-07-23).

A GE ultrasonic diagnostic instrument was used (model: Voluson E8) to measure the transvaginal three-dimensional volume. The probe frequency was adjusted between 3.0mhz and 9MHz, and the instrument has its own voice and sono AVC software package. Transvaginal ultrasound was performed prior to the follicular phase in the DOR and ovarian function Normal-groups, while the examination time of the POF-group was not limited. The patient was asked to thoroughly empty their bladder before the examination and lie in the bladder lithotomy position. A routine ultrasound examination was completed, and the condition of the uterus and endometrium were observed. Key examinations on both ovaries were completed to obtain ovarian volume, ovarian echo and follicular number.

The best quality images of the largest longitudinal section of the ovary with rich blood flow were collected and saved from repeated examinations. Offline analysis was completed using image processing software, and one of the three mutually perpendicular sections was used as the reference plane. The outline of the ovarian boundary was manually traced with each section maintaining a rotation angle of 15°. The three-dimensional ultrasound volume of the ovary was obtained with the help of post-processing software following 12 manual tracings. Then the blood flow histogram mode was used to automatically calculate VI, VFI and FI. The Sono AVC function was used to automatically select differentiated color to mark each follicle.

Three diameter lines were obtained, and the average volume and diameter of each follicle was calculated. They were arranged according to their specific diameter to ensure that the target color was related to the position of the follicle. If the boundary created by the computer was not accurate, manual modification could be completed to correct the deviation of ovarian size and count. Measurement of unilateral ovarian AFC was repeated twice, and the sum of ovarian follicles on both sides was calculated. The final results of OV, VI, VFI and FI were the average values of both ovaries.

### Sex hormone detection:

5ml of fasting venous blood was collected in the morning on the 3rd to 5th days of the menstrual cycle. Blood samples were stored at -20°C, and the levels of FSH, E2 and LH were detected by radioimmunoassay. The left and right ovary ultrasonic examination indexes (AFC, OV, VI, VFI and FI) of patients in the DOR- and POF-groups were observed. Each index is the average value of the results of repeated measurement of the unilateral ovary. The ultrasonic examination indexes (AFC, OV, VI, VFI and FI) of the DOR-group, POF-group and Normal-ovarian function group were compared. The sum of ovarian follicles on both sides was taken from AFC comparison between groups, and the average value of the ovaries on both sides was taken as the final detection result of OV, VI, VFI and FI.

A decrease in AFC, OV, VI, VFI and FI indicates the decline of ovarian reserve function. Using sex hormone test results as the gold standard, the diagnostic specificity, sensitivity and accuracy of 3D-TVS for DOR and POF were calculated. A is true positive, B is false positive, C is false negative, D is true negative. Sensitivity =A/ (A+C) ×100.0%, Specificity =D/ (B+D)×100.0%, Accuracy = (A+D) / (A+B+C+D).

### Statistical Analysis:

SPSS 22.0 was used for statistical analysis. The measurement data is represented by (*χ̅*±*S*), and the counting data is represented by n (%). A sample t-test was used to compare measurement data between groups, analysis of variance was used to compare multiple groups of measurement data, *χ^2^* was used to compare counting data is. When *p*<0.05, the difference was statistically significant.

## RESULTS

DOR-group patients ranged in age from 19-41yrs, with an average of 30.8±6.1yrs. POF-group patients ranged in age from 19-42yrs, with an average of 33.2±5.6yrs. While the Normal-group ranges in age from 20-41yrs, with an average of 31.6±5.2yrs. There was no significant difference in the average age between the three groups (*p*>0.05). There was no significant difference in AFC, OV, VI, VFI and FI between the left and right ovaries in the DOR-group (*p*>0.05), as shown in Table-I. There was no significant difference in AFC, OV, VI, VFI and FI between the left and right ovaries in the POF-group (*p*>0.05), Table-II.

**Table-I:** Comparison of ultrasonic examination results of left and right ovaries in DOR-group (*χ̅*±*S*).

Ovarian position	OV (cm^3^)	VI	VFI	FI	AFC (PC)
Left ovary (*n*=25)	4.75±1.01	1.53±0.45	0.45±0.12	27.12±3.22	6.60±2.06
Right ovary (*n*=25)	4.66±1.06	1.47±0.43	0.44±0.13	26.12±2.74	6.44±2.12
t	0.311	0.370	0.466	1.344	0.299
p-Value	0.758	0.715	0.645	0.191	0.767

**Table-II:** Comparison of ultrasonic examination results of left and right ovaries in POF-group (*χ̅*±*S*).

Ovarian position	OV (cm^3^)	VI	VFI	FI	AFC (PC)
Left ovary (*n*=32)	2.53±0.74	0.59±0.23	0.18±0.04	23.43±3.02	2.50±0.98
Right ovary (*n*=32)	2.69±0.75	0.62±0.24	0.19±0.03	23.06±3.53	2.40±0.98
t	-0.833	-0.566	-1.046	0.555	0.399
p-Value	0.384	0.575	0.304	0.583	0.693

**Fig. 1:**
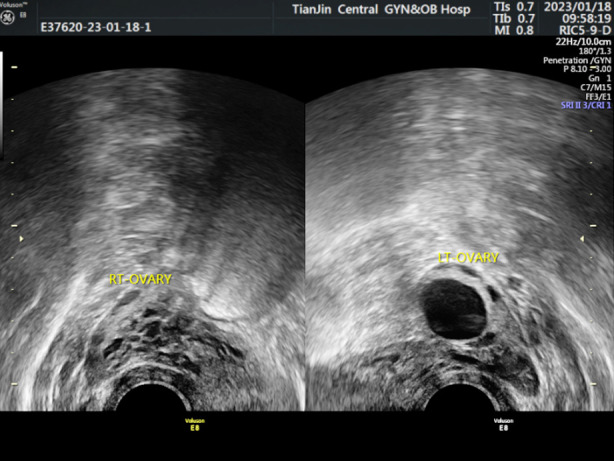
Ultrasonic examination image of Normal-group.

**Fig. 2:**
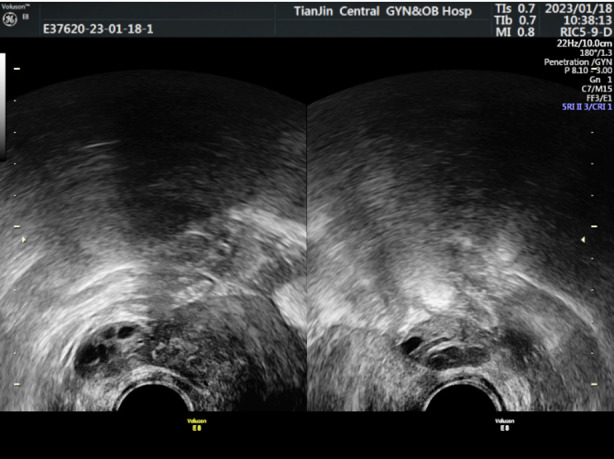
Ultrasonic examination image of DOR-group.

**Fig. 3:**
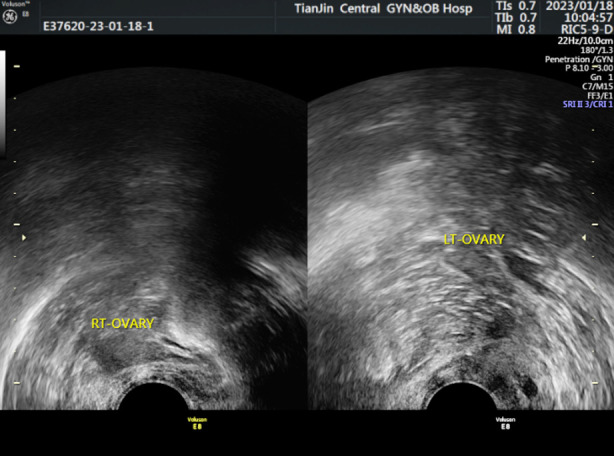
Ultrasonic examination image of POF-group.

The ultrasound examination of the Normal-group showed that the ovary was full, the echo was uniform, the sinus follicles were clear, and the ovarian blood flow was rich (Fig.1); In DOR-group, the ultrasound showed that the ovarian image was unclear, not full, and the ovarian blood flow was rich (Fig.2). In POF-group, ultrasound showed uterine atrophy, unclear image of ovary, occasional sinus follicles, and low blood flow signal (Fig.3). When compared to the Normal-group, AFC, OV, V, VFI and FI were significantly lower in both the DOR- and POF-groups (*p*<0.05). When the DOR-group was compared to the POF-group, these indexes were significantly lower (*p*<0.05). Table-III.

**Table-III:** Ultrasonic examination results of left and right ovaries in the DOR-group, POF-group and Normal ovarian function group (*χ̅*±*S*).

Group	OV (cm^3^)	VI	VFI	FI	AFC (PC)
DOR-group (*n*=25)	4.71±0.69^a^	1.50±0.17^a^	0.44±0.08^a^	26.62±2.34^a^	13.04±3.22^a^
POF-group (*n*=32)	2.55±0.53^ab^	0.59±0.15^ab^	0.18±0.03^ab^	23.25±2.68^ab^	4.90±1.44^ab^
Normal-group (*n*=63)	7.39±2.22	2.81±0.61	0.89±0.24	31.08±3.17	19.54±4.41
F	132.812	210.211	230.280	100.149	187.265
*p-Value*	<0.001	<0.001	<0.001	<0.001	<0.001

***Note:*** a indicates a comparison with the Normal-group, p<0.05; b indicates a comparison with the DOR-group, p<0.05.

Using the sex hormone test results as the gold standard, the diagnostic specificity, sensitivity and accuracy of 3D-TVS for DOR and POF were calculated. The results showed that the diagnostic specificity for DOR was 80.00%, the sensitivity and accuracy were 90.00% and 88.00% respectively. The diagnostic specificity of POF was 87.50%, and the sensitivity and accuracy were 95.83% and 93.75% respectively. For the specific diagnostic results.Table-IV.

**Table-IV:** Diagnostic results of DOR and POF by transvaginal three-dimensional ultrasound.

Group	n	True positive (n)	False positive (n)	False negative (n)	True negative (n)	Accuracy (%)	Specificity (%)	Sensitivity (%)
DOR-group	25	18	1	2	4	88.00	80.00	90.00
POF-group	32	23	1	1	7	93.75	87.50	95.83

## DISCUSSION

This study investigated the application value of 3D-TVS in the assessment of DOR and POF, with the results of sex hormone detection as the gold standard, the specificity, sensitivity and accuracy of 3D-TVS in the diagnosis of DOR were 80.00%, 90.00% and 88.00% respectively; The specificity of POF was 87.50%, the sensitivity and accuracy were 95.83% and 93.75% respectively. Wu J et al^9^ showed through a case-control study (308 cases vs 300 cases) that transvaginal ultrasound is of great value in evaluating ovarian reserve function and predicting ovulation, with specificity higher than 90.00% and sensitivity higher than 80.00% for AFC and OV. This is consistent with the results of this study. In addition, the results of this study showed that there was no significant difference in the levels of AFC, OV, VI, VFI and FI between the right and left ovaries of patients in DOR and POF-groups (*p*>0.05). While DOR and POF may not cause significant differences in ovarian function between the left and right sides, some studies have found that the OV of the right ovary in patients with DOR at the early follicular stage is significantly larger. Additionally, the levels of AF, VI, VFI and FI can also be significantly higher, a result which is different from those of this study.^11^ These difference may be due to the fact that the inflow site of the right ovarian vein is the inferior vena cava, while the inflow site of the left ovary is the left renal vein.^11^,^12^ Currently, there are few clinical comparative analyses of the left and right ovaries using three-dimensional ultrasound, and further discussion is warranted.^13^,^14^ Our results reported lower AFC, OV, VI, VFI and FI within both the DOR-group and the POF-group versus the Normal ovarian function group (*p*<0.05). These indexes were also lower within the POF-group than the DOR-group (*p*<0.05). This is consistent with the clinical results of Karlberg S et al^15^ and Igboeli P et al^16^, suggesting that the weaker the ovarian reserve function of mature women, the less their OV and AFC will be paralleling a gradual decline in VI, VFI and FI. Transvaginal three-dimensional ultrasound technology can help identify and diagnose DOR and POF in the early stage.^15^,^16^ This technique can conduct scientific and accurate quantitative analysis on the three-dimensional morphological changes of ovaries and follicles, to provide more effective reference information for the clinical diagnosis of DOR and POF.^17^,^18^

In the past, the hormone level was mainly detected by biochemical examination in the evaluation of ovarian reserve function, but the biochemical examination had certain limitations and could not obtain the morphological information of the ovary.^9^,^11^ Vaginal ultrasound is the best way to monitor follicles and the most accurate method at present.^9^ Through the use of post-processing software, list marking processing can be implemented for different follicle diameters and volumes, which is convenient for clinicians to observe the subtle follicular structural changes and improves analysis of sinus follicle development. Yu L et al^12^ discussed the application value of transvaginal color Doppler ultrasound based on improved average shift algorithm in the diagnosis of idiopathic POF, the results show that transvaginal color Doppler ultrasound based on artificial intelligence segmentation algorithm can clearly show the functional state and hemodynamics of the patient’s egg nest. This technique has high repeatability, which can effectively reduce subjective error and improve diagnostic accuracy.^16^ This study shows that transvaginal three-dimensional ultrasound has good diagnostic efficacy for both DOR and POF, which is consistent with the results of Singhal P et al.^19^ The use of 3D-TVS technology in the diagnosis and evaluation of DOR and POF does have application value.^15^,^18^ With the progress and update of the examination equipment, ultrasound technology is simple, fast, non-invasive and accurate, and has been gradually cited in clinical work. Three dimensional ultrasound complements the deficiencies of two-dimensional ultrasound in providing quantitative indicators and ovarian quantitative volume.^20^ It can quantize ovarian imaging evaluation indicators and make ovarian volume more intuitive and accurate. 3D-TVS provides valuable scientific basis and reference information for clinical treatment.^21^

### Study limitations:

This is a retrospective single center analysis, and the number of patients included is relatively small. Due to time and other factors, it was not possible to carry out follow-up observation on the patients, and therefore it was not possible to further understand the pregnancy and subsequent ovarian function changes of patients in each group. Future examinations should further expand the sample size and include follow-up observations to further confirm the application value of this examination method.

## CONCLUSION

These results support the use of 3D-TVS in the clinical diagnosis and evaluation of DOR and POF. As early diagnosis and optimized treatment of DOR is the key to limiting the development of POF, the utilization of 3D-TVS could be an important component of examination for these patients. Future analysis of larger clinical studies, as well as longer follow-up examinations can further enhance the clinical application of this technique.
